# The road to change: Broadband China strategy and enterprise digitization

**DOI:** 10.1371/journal.pone.0269133

**Published:** 2022-05-31

**Authors:** Chao Wang, Man Zhang

**Affiliations:** College of Business, Shanghai University of Finance and Economics, Shanghai, China; Al-Balqa Applied University Prince Abdullah bin Ghazi Faculty of Information Technology, JORDAN

## Abstract

The digitization of a company necessitates not only the effort of the company but also state backing of network infrastructure. In this study, we applied the difference-in-differences method to examine the impact of the Broadband China Strategy on corporate digitalization and its heterogeneity using the data from Chinese listed firms from 2010 to 2020. The results show that the improvement in network infrastructure plays a vital role in promoting company digitization; this improvement is extremely varied due to variances in market demand and endowments. Non-state-owned firms, businesses in the eastern area, and technology-intensive businesses have profited the most. Among the five types of digitization, artificial intelligence and cloud computing are top priorities for enterprises. Our findings add to the literature on the spillover effects of broadband construction and the factors affecting enterprise digitalization.

## Introduction

Large changes have occurred in China since the economic reform in 1978. According to the National Bureau of Statistics, China surpassed Japan to become second largest economy in the world. However, as Krugman (1994) stated, the "Asian miracle" is essentially fueled by labor rather than inspiration [1]. With the rise in labor expenses and the tightening of environmental rules, China’s GDP growth has fallen from double digits to 6% in 2019. As a result, the Chinese government is banking on new economic development through intelligent industrial transformation and proposed the national construction of network infrastructure through the Broadband China Strategy (BCS) in 2013. The development of the digital economy has become urgent and essential, especially after the COVID-19 pandemic when telecommuting, automated manufacturing, and big data sales have all played major roles in increasing corporate efficiencies.

Nevertheless, for most companies, digital transformation still presents many challenges. A McKinsey survey reported that the failure rate of corporate digitization is as high as 80%, and more than 55% of companies have not even completed basic digital transformation. Enterprise digitization is essentially a type of technological change and innovation, which explained by Solow’s paradox, has a positive externality. When one company uses digital technology to improve its own efficiency, others may profit as well. For example, when corporations employ robots to manufacture their products, they not only increase their market share but also cut the input costs of downstream industries. Classical researchers assumed that in the presence of positive externalities, product supply is inadequate. This also explains why corporations benefit from theoretical digitization whereas many companies underinvest in reality. Furthermore, the problem of coordination failure persists in digitalization. With the growth in corporate digitalization, efficiency gains occur through cooperation rather than division of labor. If upstream and downstream industries do not cooperate to undertake digital transformation, efficiency will decrease rather than rise. For instance, if one company wants to digitally manage the supply chain and ensure the traceability of product information, while its suppliers have no interest in digitalization and prefer the old processes, then cooperation will be difficult. Based on the above analysis, we draw the straightforward conclusion that the externality of digitization and coordination failure might cause enterprises to underinvest. As Keynesian Economics state, government provides a crucial supplement to avoiding market failure. Therefore, our the primary concern in this study was to determine the role of government in enterprise digitalization.

The BCS proposed by the Chinese government in 2013 has played a beneficial facilitating role in enterprise digitization. This strategy aims to improve network penetration and service quality. Its specific goals was that by 2015, more than half of households would have access to the Internet with a least access capacity of 20 Mbps, and 100 Mbps in some developed cities; by 2020, the broadband network would be fully covered, and the penetration rate would reach 70%. As for enterprises, the Chinese government aimed to boost business network speeds to 100 Mbps in 2015 and 1000 Mbps in 2020. The following policy measures have been implemented: First, coordinated regional broadband network development was promoted, the optimization and upgrading of existing broadband networks have been expedited, and network speed and reliability have been improved. Second, the quality of broadband network applications has been improved and enterprises have been encouraged to upgrade their network and intelligence levels. Third, digital industries have been developed, such as cloud computing and big data. Fourth, the use of broadband networks has been expanded to education, medical care, employment, and social security.

The Chinese government expects that the strategy will be successful on both the supply and demand sides of the information industry. This massive broadband infrastructure construction has improved the conditions and speed of the national network, thereby increasing the supply of digital products and services; it also provides convenience for enterprises to apply new technologies such as cloud computing and big data. BCS has increased the digital application scenarios for 1.4 billion Chinese people, which in turn stimulated the enthusiasm of enterprises for digitalization from the demand side.

The rest of the paper is organized as follows: Section 2 provides a review of the related literature. Section 3 describes the difference-in-differences (DID) model and data. Section 4 describes the regression results, robustness checks, and heterogeneity exams; Section 5 outlines our conclusions.

## Literature review

The literature related to our study can be divided into two categories. The first type involves examinations of the spillover effects of broadband infrastructure facilities. Building broadband networks, compared with traditional infrastructure, is faster and requires less space and has important network effects and externalities. Building network infrastructure boosts total factor productivity [2]. The spillover impacts of broadband development has been analyzed from several aspects. Some researchers focused on the relationship between broadband access and regional employment [3–5], finding that the deployment of broadband increased employment in the service industry and increased rural wages [6]. Conversely, the availability of remote work may decrease regional employment [7]. Canzian et al. (2019) internet availability in the Trento region of Italy as a natural experiment and found that broadband access helped to increase corporate productivity [8]. More knowledge-intensive businesses are attracted by broadband, resulting in an agglomeration effect [9]. Wolfgang et al. (2021) studied 401 German counties from 2010 to 2015 and discovered that the deployment of broadband not only produced huge economic benefits in terms of direct impact inside the counties, but also produced favorable regional externalities across counties [10]. According to Wolfgang’s calculation, for every unit (1 Mbit/s) increase in the average bandwidth speed, the regional GDP increases by 0.18%. In the context of global warming, some researchers have paid attention to the impacts of broadband access on energy conservation and emission reduction. Based on a survey of 196 cities in 30 Chinese provinces, Wu et al. (2021) found that the spread of the Internet has supported energy conservation and emission reduction through technical progress, energy structure, human capital, and openness [11]. The macro-level spillover effects of broadband deployment have been thoroughly examined. The environmental performance of manufacturing businesses has substantially improved as a result of the industrial digital transformation process, with structural and technology impacts as the transmission channels [12]. However, the implications of broadband facilities on micro-level firms in China remain unknown.

The second body of literature related to our study mainly focuses on enterprise digitization. In the new round of global industrial competition, the digital economy has become an important force driving industrial upgrading and economic growth. Governments of all countries must speed up the digital upgrade of traditional industries, and integrate the digital and the real economies. Especially after the outbreak of COVID-19, the new economy, represented by cloud shopping, online medical care, and distance education, will not only improve the daily lives of residents but also reduce the negative impacts of potential supply chain disruptions [13]. The digital economy, with digital industrialization and industrial digitization at its core, has become a new major change in the world following the industrial and the information revolutions [14]. However, the digitization of firms, particularly in developing nations where digitalization acceptance is low, is the key impediment to the growth in the worldwide digital economy [15]. Due to various reasons, some companies in China are unwilling to digitize, refuse to digitize, or do not know how to digitize. Researchers have mostly concentrated on the influence of digital transformation on business performance. For example, Finkelstein et al. (2021) found that the company’s digital adoption rate has a significant negative relationship with the self-employment rate, but no significant impact on the unemployment rate [16]. Scott (2017) studied 6848 banks in 29 countries and regions in Europe and the Americas, and found that the adoption of digital innovation has a huge and positive long-term impact on bank performance; as the number of adopters increases, the network effect contributes to the performance of the bank, and not vice versa [17]. Forman (2019) focused on the impact of digital technology adoption on the cross-regional flow of knowledge [18], and found that the adoption of the Internet increased the possibility of citation between paired R&D locations within the company. This effect was mainly achieved when both places had access to the Internet. Tranos (2020) reported that the creation of online content in 2000 had a significant positive and lasting impact on regional productivity for the next 16 years [19]. In summary, most of these researchers found that the digital economy, especially the implementation of digitalization, positively impacts both economic and social development. However, only a few researchers examined the elements determining corporate digitalization. For example, Zhu et al. (2006) found that compatibility and security are key factors in enterprises adopting digital technology, and their role even exceeds their cost [20]. Ananda (2020) reported that awareness, network functions, and perceived usefulness have a significant positive impact on the adoption of digital banks [21]. Overall, little is known about the forces driving enterprise digitalization, especially the causality between telecommunications infrastructure construction and enterprise digitization.

The digital economy has emerged as a critical factor driving countries to improve their long-term competitiveness and achieve green development [22]. Nevertheless, in the first decade of the 21^st^ century, the level of digital transformation of enterprises was low in China due to the lack of external support. The prompt implementation of BCS provided the essential network infrastructure support, alleviating business worries and broadening the scenarios in which digitization could be applied. Drawing from the two sets of literature, we found that studies on the spillover effects of broadband construction mostly concentrated on regional or sector indicators, such as employment and industrial structure, whereas few have considered the impact on direct corporate digital operations. Furthermore, previous researchers primarily focused on the after effect of enterprise digitization, yet overlooked the forces driving digitization.

In this study, we applied the panel data of companies listed in the Shanghai and Shenzhen stock market from 2010 to 2020 to empirically examine the impact of the BCS on corporate digitalization and its heterogeneous characteristics. Our findings provide four contributions: (1) Starting from the externality characteristics of enterprise digitalization, we analyzed the important of government construction of network facilities and supplement the research on the externalities of broadband construction and the forces driving enterprise digitalization, which has theoretical and practical significance. (2) Digital industrialization and industrial digitization are important components of the digital economy. Our findings show that the digital industry has a good enabling effect on industrialization and should be promoted in a coordinated manner. (3) The digitalization of enterprises is heterogeneous; thus, in this study, we distinguished the impact of broadband construction from the perspectives of geographic location, ownership, and element characteristics. In addition, we also evaluated the impact of the BCS on different kinds of digitalization and found that among five types of digitalization, artificial intelligence and cloud computing are the most affected sources. (4) Broadband construction and enterprise digitization have reverse causality. We used geographic fluctuations as an instrumental variable to further verify this potential deviation, making the research results more credible.

## Econometric model and data

### Model specification

Economists are often concerned with the effects of policy implementation, for example, the effect of minimum wage on employment [23] and the effect of state abortion laws on teen pregnancy [24]. The easiest method of determining these effects is to compare the before and after differences between the treatment groups (i.e., the regions or individuals affected by the policy). This is called a differential estimator, which calculates the sample mean of the group after the policy was implemented minus the sample mean before the policy was implemented. However, because the macroeconomic environment also changes over time, differences before and after policy implementation may not necessarily indicate the effect of a treatment. Therefore, in the double-difference method, the areas where the policy was not implemented are taken as the counterfactual reference of the treatment group, and the changes before and after the treatment group are subtracted from the changes before and after in the control group, producing a more reliable estimate of the policy treatment effect. This method was first introduced into economics by Ashenfelter in 1978 [25], and was later popularized by Card [26], Abadie [27], Angrist [28, 29], Imbens [30], Duflo [31, 32], and Qian [33]. It has become one of the most important methods to determine causal inference. The 2021 Nobel Prize in Economics was awarded to Angrist and Imbens for their contribution to the methodology of causal analysis.

The four setup requirements when using DID for policy evaluation are as follows: First, the policy cannot be a one-size-fits-all type, that is, an experimental group that is affected by the policy and a control group that is unaffected by the policy. The BCS is a pilot policy, which implies that some cities implemented this policy, whereas others did not. Second, the treatment and the control groups should satisfy the parallel trend assumption: before the policy intervention, the trends in the outcome effect of the treatment and control groups should be the same. Third, the policy intervention only affects the treatment group and does not have an interactive effect on the control group, or the policy intervention will not have spillover effects. Finally, the potential outcome, processing, and the time variables must satisfy the linear condition. The specific form of our model is as follows:

$$lndig_{i,t}=\beta_{0}+\beta_{1}Treat_{c}\times Post_{t}+\beta_{2}Treat_{c}+\beta \times \sum Z_{i,t}+\mu_{i}+\tau_{t}+\epsilon_{i,t)}$$ (1)

where *lndig_i,t_* is the dependent variable that indicates the digital level of enterprise i in year t; *Treat_c_*×*Post_t_* is the core explanatory variable of this DID model, where *Treat_c_* represents whether the city c in which the company i is located has implemented the BCS. If city c is on the list, then *Treat_c_* = 1; otherwise, *Treat_c_* = 0. *Post_t_* represents whether the observation sample is located after or before the implementation of the policy. If *Post_t_* = 1, the policy has been implemented; otherwise, the value is 0. As enterprise digitalization may be affected by other characteristics, we also controlled a series of variables, including company age, capital intensity, growth, total assets, executive digital background, and return on assets. We included the fixed effects of the company *μ_i_* and the year *τ_t_* to address the issue of missing variables. Finally, we included the random error term *ϵ_i,t_* in the model.

### Parallel trend test

An assumption of the DID model is parallel trends, that is, before the policy is implemented, no significant difference existed between the digitalization of listed companies located in pilot cities and nonpilot cities. We referred to the practices of Beck et al. [34] and used the event analysis method to construct the following dynamic model:

$$lndig_{i,t}=\alpha_{0}+\sum_{m=-4}^{-1}\theta_{m}Pre_{m}+\sum_{n=1}^{6}\theta_{n}Post_{n}+\beta_{2}Treat_{c}+\beta \times \sum Z_{i,t}+\mu_{i}+\tau_{t}+\epsilon_{i,t}$$ (2)

where *lndig_i,t_* represents the digitization level of the listed company; $\sum_{m=-4}^{-1}\theta_{m}Pre_{m}$ represents a set of counterfactual dummy variables, that is, the dummy variables from the 1st to 4th years before the implementation of the BCS; $\sum_{n=1}^{6}\theta_{n}Post_{n}$ is the dummy variable from the 1st to the 6th year after the implementation of the policy. To be consistent with Eq (1), we added control variables and fixed effects. If the regression coefficient *θ_m_* does not pass the significance test, the firm digital level did not significantly differ between the experimental group and the control group before the implementation of the policy; if *θ_n_* passes the significance test, the BCS had a causal effect on firms’ digital investment, and passed the parallel trend test.

### Variables and data

(1) Explained variable. *lndig_i,t_* is the level of digitization of the enterprises. To be consistent with typical academic standards, we used data from the CSMAR database. The most commonly used identification of firm-level digitization is the corresponding keyword frequency in the annual report of listed companies. The CSMAR database is divided into five categories according to keywords and digital characteristics: artificial intelligence, big data, blockchain, cloud computing, and digital technology applications. The keywords corresponding to these digital indicators are listed in Table 1. Because these data have typical right-biased characteristics, we conducted logarithmic processing to obtain an index that characterizes the level of enterprise digitalization.

**Table 1:** Keywords for digital measurement.

Digital Type	Key words
Artificial Intelligence	Machine learning, artificial intelligence, face recognition, business intelligence, authentication, deep learning, Biometrics technology, image understanding, semantic search, speech recognition, intelligent robot, intelligent data analysis, autopilot, natural language processing (14)
Big Data	Mixed reality, data visualization, data mining, text mining, virtual reality, heterogeneous data, augmented reality, credit reporting (8)
Cloud Computing	EB level storage, multi-party secure computation, neuromorphic computing, streaming computing, green computing, memory computing, cognitive measurement, fusion architecture, graph computing, Internet of things, cyber-physical system, exascale concurrence, cloud computing (13)
Blockchain	Bitcoin, distributed computing, consensus mechanism, consortium blockchain, decentration, digital currency, smart contract (7)
Digital Technology and Application	B2B, B2C, C2B, C2C, Fintech, NFC payment, O2O, third party payment, e-commerce, industrial Internet, Internet finance, Internet healthcare, financial technology, open banking, quantitative finance, digital finance, digital marketing, netsunion, cashier-less retail, mobile interconnection, mobile Internet, mobile payment, intelligent agriculture, wearable smart devices, smart grid, smart environment, home automation, intelligent transportation, intelligent contact center, intelligent energy, robo-adviser, intelligent culture tour, intelligent healthcare, intelligent sales and marketing (34)

(2) Core explanatory variables. We used the BCS to represent the construction of network facilities. We obtained the list of pilot cities from the official website of the Ministry of Industry and Information Technology of China. To date, the BCS has identified three batches of pilot cities (2014, 2015, and 2016). Because certain municipalities are special provincial-level administrative divisions directly under the reign of the Central Government, we omitted four municipalities, Beijing, Shanghai, Tianjin, and Chongqing, from the list and finally considered 116 pilot cities (experimental group) and 169 nonpilot cities (control group). The statistics of the selected provinces are shown in Table 2, where we use different colors to differentiate four districts in China. Green indicates the eastern seaside region, blue represents middle areas, orange represents western district, and gray represents the northeast region. The selection of pilot cities was relatively balanced.

**Table 2:** BCS pilot cities.

Province	2014	2015	2016	Province	2014	2015	2016
Hebei	Shijiazhuang			Shaanxi			Weinan
Shandong	Qingdao	Dongying	Yantai	Sichuan	Chengdu	Mianyang	Ya’an
	Zibo	Jining	Zaozhuang		Panzhihua	Neijiang	Luzhou
	Weihai	Dezhou			Aba	Yibin	Nanchong
	Linyi					Dazhou	
Jiangsu	Nanjing	Yangzhou	Wuxi	Yunnan		Yuxi	Wenshan
	Suzhou		Taizhou	Guizhou	Guiyang		Zunyi
	Zhenjiang		Nantong	Guangxi			Yulin
	Kunshan			Gansu		Lanzhou	Wuwei
Zhejiang	Jinhua	Jiaxing	Hangzhou			Zhangye	Jiuquan
Guangdong	Guangzhou	Shantou					Tianshui
	Shenzhen	Meizhou		Qinghai			Xining
	Zhongshan	Dongguan		Ningxia	Yinchuan	Guyuan	
Fujian	Fuzhou	Putian			Wuzhong	Zhongwei	
	Xiamen			Xizang			Lhasa
	Quanzhou						Linzhi
Hainan			Haikou	Xinjiang	Alar	Karamay	
Shanxi		Taiyuan	Yangquan	Inner Mongolia		Hohhot	Wuhai
			Jinzhong			Ordos	Baotou
Henan	Zhengzhou	Xinxiang	Shangqiu				Tongliao
	Luoyang	Yongcheng	Jiaozuo	Liaoning	Dalian	Anshan	Shenyang
			Nanyang		Benxi	Panjin	
Anhui	Wuhu	Hefei	Suzhou	Jilin	Yanbian	Baishan	
	Anqing	Tongling	Huangshan	Heilong jiang	Harbin		Mudan jiang
			Maanshan		Daqing		
Hubei	Wuhan	Huangshi	Ezhou	Jiangxi	Nanchang	Xinyu	Ji’an
		Xiangyang			Shangrao	Ganzhou	
		Yichang		Hunan	Changsha	Yueyang	Hengyang
		Shiyan			Zhuzhou		Yiyang
		Suizhou			Xiangtan		

(3) Control variables. To improve the research accuracy, in the regression model, we also controlled a series of company characteristic variables that could have affected the company’s digital level [35]: company age (age, logarithmic processing), capital intensity (sd, the ratio of total assets to operating income), growth index (grow, total asset growth rate), corporate total assets (lnass, logarithmic processing), executive digital background (ceo_dig, if any of the listed company executives has a digital background, then it takes a value of 1; otherwise, it is 0), and return on assets (roa, net profit/total assets). Except for the digital background of executives, other control variables have often been used in studies. According to McKinsey’s survey report, corporate digitization is an executive project. The digital strategy must be implemented from top to bottom. We think that if executives have a digital education background, they will not only encourage digital transformation but also better lead digital transformation. We regarded an educational background involving information, intelligence, software, electronics, communications, communications, systems, networks, automation, wireless, and computers as executives with a digital background. A detailed description of the variables is provided in Table 3.

**Table 3:** Descriptive statistics of variables.

Variable	N	Mean	SD	Min	Max	Treat = 0	Treat = 1	T-test
lndig	21295	0.987	1.270	0.000	6.114	0.739	1.104	-0.365***
lnage	21295	2.846	0.360	0.693	4.143	2.849	2.844	0.005
sd	21295	2.501	2.179	0.414	15.69	2.439	2.531	-0.091***
grow	21295	0.177	0.352	-0.352	2.318	0.167	0.182	-0.015***
lnass	21295	22.09	1.234	16.160	28.26	22.09	22.09	0.006
ceo_dig	21295	0.054	0.225	0.000	1.000	0.031	0.064	-0.032***
roa	21295	0.043	0.071	-0.280	0.782	0.041	0.043	-0.002**

Note

*, **, and *** indicate 10%, 5%, and 1% significance levels, respectively.

Data source: authors’ calculations.

The digitalization of the treatment group implementing the BCS was substantially higher than that of the control group. This preliminarily showed that the BCS may have promoted the digitization of enterprises. However, the characteristics of the treatment group enterprises are notably different. This means that a simple comparison of the two sets of digitization may be biased, and so empirical analysis was required while controlling for other factors.

This article selected the Shanghai and Shenzhen A-share listed companies from 2010 to 2020 as a sample, and processed the data as follows: first, we removed the sample of terminated listed companies and warning firms such as ST, *ST, and PT. ST denotes special treatment; PT denotes particular treatment. These companies have suffered heavy losses and are on the verge of being delisted.; second, we removed financial service companies; third, to reduce interference by outliers, we tailed down 1% and 99% of continuous variables.

## Results and discussion

According to the model setting, we first performed a benchmark regression to examine whether the BCS promoted enterprise digitization. Then, we examined a series of robustness tests and endogenous treatments. Finally, we obtained the results.

### Benchmark regression

To verify that the variables showed no multicollinearity, the results of our pairwise correlation are presented in Table 4. The results showed that the correlation coefficients between the variables are all lower than 0.3, indicating a lack of multicollinearity.

**Table 4:** Pairwise correlation.

Variables	lndig	lnage	sd	grow	lnass	ceo dig	roa	Treat×Post
lndig	1							
lnage	0.033***	1						
sd	-0.049***	0.077***	1					
grow	0.030***	-0.089***	0.059***	1				
lnass	0.059***	0.192***	-0.005	0.076***	1			
ceo dig	0.185***	-0.063***	-0.003	0.027***	-0.012*	1		
roa	0.002	-0.056***	-0.194***	0.209***	0.061***	0.004	1	
Treat×Post	0.279***	0.218***	0.027***	-0.009	0.094***	0.056***	0.008	1

Table 5 shows the benchmark regression results of the influence of BCS on enterprise digitization. Column (1) only contains the core explanatory variable Treat×Post and Treat. The results showed that network infrastructure construction significantly improved the digitalization level of local enterprises. This promotion effect was approximately 11.31%, which is significant at the 1% level. Column (2) further incorporates six control variables. The coefficient of the core explanatory variable Treat×Post decreased from 0.1131 to 0.1004, but still remained highly significant. This could be attributed to the unobservable impact of the company’s digital transformation being absorbed after the control variables were included. Benchmark regression showed that, while keeping other factors unchanged, the construction of broadband infrastructure strongly drives the digital transformation of enterprises. In both models, we controlled for the fixed effects of company and year.

**Table 5:** Regressions of the enterprise digitalization on BCS.

	(1)	(2)
	lndig	lndig
Treat×Post	0.1131***	0.1004***
	(4.1436)	(3.8541)
Treat	0.0017	-0.0247
	(0.0287)	(-0.4444)
lnage		0.3949**
		(2.2946)
sd		-0.0192***
		(-3.2454)
grow		-0.0345**
		(-2.1065)
lnass		0.2412***
		(11.3285)
ceo_dig		0.0282
		(0.7078)
roa		-0.3488***
		(-3.4052)
_cons	0.3253***	-5.7194***
	(4.2302)	(-7.8292)
*N*	21295	21295
*R* ^2^	0.2657	0.2820
FirmFE	YES	YES
YearFE	YES	YES
F	78.6285	75.1473

Note: T-statistics for each coefficient are provided in parentheses.

*, **, and *** indicate 10%, 5%, and 1% significance levels, respectively.

Data source: authors’ calculations.

The regression results for the control variables are basically in line with the expectations and economic theory. The age of the company, scale of assets, and digital background of executives positively affected the digital level of the company. This shows that compared with young companies and small companies, mature and large firms have a higher level of digitalization. The various endowments required for the digital transformation of these companies are more complete. Additionally, with increases in age and scale, companies are highly motivated to maintain a sustainable competitive advantage through digitalization. Although the digital background of executives had a positive effect, it was not significant. This may be related to the ownership structure of listed companies in China. State-owned group, and family companies still account for the majority, inhibiting the influence of senior management team such as the CEO and CFO on the company’s decision making [36]. Among the other control variables, asset intensity, growth, and return on assets significantly negatively correlated with the level of corporate digitalization. This result is in line with our intuition. The asset intensity reflects the relative degree of reliance on capital in the production and operation processes of the enterprise. The higher the asset intensity, the higher the investment risk and the higher the requirements for production technology and management level. Digitization is characterized by a high degree of uncertainty, so capital-intensive companies are often unwilling to undertake digital transformation. In addition, companies with better operational conditions have a considerably lesser inclination to digitize, despite digitization being a key strategy for increasing corporate efficiency and accomplishing transformation. Finally, the coefficient of Treat was not significant, which showed that after controlling for a series of factors, digitization in the treatment and the control groups was not significant different. Only after adding the post variable reflecting the implementation of the BCS did the two significantly differ. We next conducted the test of parallel trends, as described below.

### Robustness check

**Fig 1:**
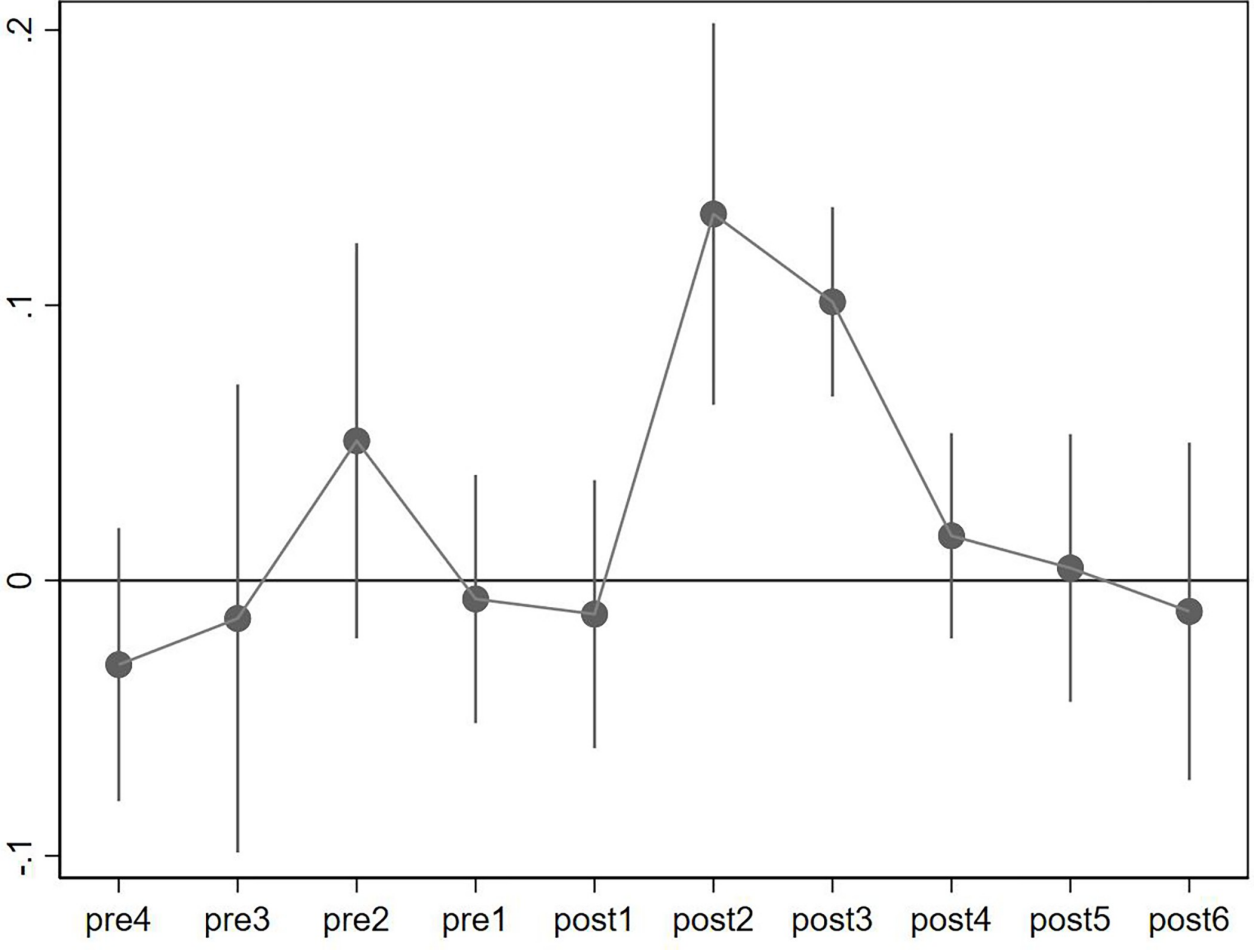
Parallel trend test.

The results of the benchmark regression showed that the implementation of the BCS has promoted the digitalization of enterprises in the pilot cities. Next, we conducted a series of robustness analyses to ensure that our conclusions are credible. First, we conducted a parallel trend test to determine the existence of any significant differences between the two sets of city samples before the implementation of the BCS. Then, we performed regression according to the setting of model (2), and plot the coefficients in Fig 1. The figure shows that before the implementation of the BCS, the digitalization of the treatment and the control groups was not significantly different. However, after the implementation of the policy, the digitalization of the treatment group enterprises considerably increased. We discovered that this promotion impact only lasted two years, implying that network infrastructure is merely a prerequisite for business digitalization. Due to the saturation of network facilities, boosting company digitalization may necessitate initiatives from other sources.

An assumption of the application of the DID method was that the selection of pilot cities in the BCS was random [37–40]. However, as stated in the policy document, the construction of broadband in China should "make full use of the existing network infrastructure", which means that the choice of pilot cities may not have been completely exogenous. To overcome potential endogenous difficulties, we employed instrumental variables. Referring to the practice of Liu and Ma [41], we used the urban topographic undulation, namely the standard deviation of each city’s specific altitude, as an instrumental variable for the BCS. We obtained these data from the Chinese Topographic Undulation Kilometer Grid Dataset constructed by You et al. [42], which we downloaded from the Global Change Research Data Publishing System. Topographic undulation satisfied the two requirements of instrument variables. The degree of topographical undulation impacts the development of digital infrastructure. The larger the degree of undulation, the worse the signal quality of the broadband network and the higher the construction costs, which impacted the network infrastructure’s operational efficiency and indirectly affected the choice of BCS pilot cities. Therefore, the terrain undulation degree as an instrumental variable satisfied the correlation condition. As a natural geographic variable, topography does not directly affect the digitalization of enterprises, so the only possible influence channel is broadband construction. Therefore, topographic undulation as an instrumental variable satisfied the exogenous conditions.

Table 6 shows the results of regression using instrumental variables. Columns (1) and (2) show the two-stage least square results using terrain undulation as an instrumental variable. The results of column (1) shows that a significant negative relationship exists between the degree of geographic fluctuation and the implementation of the BCS, which shows that the likelihood of a city being designated as a pilot city decreased as the city’s geographic undulations increased. The results of column (2) show that the BCS significantly positively impacted the level of corporate digitalization, and the coefficient was significantly increased compared with the benchmark regression results from 0.1004 to 0.3843. This finding demonstrates that, after correcting for the endogenous problem, the building of digital infrastructure still had highly favorable impacts on the level of corporate digitalization, and the degree of the effect was strongly enhanced. The F-values of the instrumental variables in the first stage were much later than 10, indicating that the instrumental variables were not weak. Our conclusion that the creation of digital infrastructure has increased the digital level of companies is valid.

**Table 6:** Results of IV and robustness check.

	(1)	(2)	(3)	(4)
	Treat×Post	lndig	lndig	lndig
Treat×Post		0.3843**	0.1013***	0.0751***
		(2.1308)	(3.9381)	(2.8859)
geo	-0.0870**			
	(-2.1871)			
lndesub			0.0146***	
			(5.6911)	
lnict				0.0784***
				(3.6364)
lngdp				0.0007
				(0.0168)
*N*	20920	18290	21295	18307
*R* ^2^	0.5826	0.0209	0.2839	0.2572
Control	YES	YES	YES	YES
FirmFE	YES	YES	YES	YES
YearFE	YES	YES	YES	YES
F	372.0635	62.7854	70.0783	52.4931

Note: T-statistics for each coefficient are in parentheses.

*, **, and *** indicate 10%, 5%, and 1% significance levels, respectively.

Data source: authors’ calculations.

We implemented another two robustness tests and the results are shown in columns (3) and (4). First, in emerging economic countries such as China, government subsidies, that is, the central or local governments guiding companies to invest in digital construction through special grants or tax reductions and exemptions, are a common method of influencing corporate decision making. We constructed the control variable lndesub to reflect the subsidies received by the digital industry at the enterprise level. Our specific lndesub calculation process was as follows: First, we identified companies engaged in the digital industry based on whether the main business of the listed company contained digital keywords. Second, using Baidu API to obtain the city where the company’s office was located, we used the asset-weighted method to calculate the average amount of subsidies received for digital industrialization in that city. Finally, considering the right-biased characteristics of the data, we conducted logarithmic processing to obtain an index that characterized the subsidy intensity of digital industrialization. The regression findings revealed that after adjusting for the influence of government subsidies, the conclusion that digital infrastructure improved the degree of business digitalization remained robust. The coefficients of the core variables are close to the benchmark regression, and we found that the newly added control variable lndesub was significant at the 1% level. This also demonstrated that government subsidies stimulated enterprise digitalization; therefore, we verified the legitimateness of this robustness test.

The second robustness test considered city-level factors, such as the number of information and communication technology (ICT) employees and per capita GDP. We obtained the data from the *China City Statistical Yearbook* for each year. Although the above test proved that the construction of digital infrastructure significantly promoted the digitalization of enterprises, the potential competitive explanation is that a more-developed information industry in a city could have promoted the digitalization of enterprises. Therefore, we used the proportion of the city’s ICT industry employees to the total labor force to control this factor. In addition, the level of economic development of the city may have stimulated companies to digitize. We used the city’s current GDP to control this factor. From the results of column (4), even after controlling the development of the urban ICT industry and the level of economic development, we found that the coefficients of the core explanatory variables were still significantly positive, confirming the robustness of our conclusions from benchmark regression.

### Heterogeneity analysis

Next, we examined whether the influence of network infrastructure on the level of digitalization of enterprises differed due to the geographical location, nature of the enterprise, factor intensity, or digital sources.

(1) Subgroup based on the three regions. Due to historical and natural reasons, China’s urban development has differed in different areas. For example, in general, the eastern region has a more developed urban economy, with a higher proportion of tertiary industry. The central region shows average economic performance, with a higher proportion of secondary industry; the western region has a poorer natural environment and low population density. Therefore, we identified companies located in eastern, central, and western cities according to the classification of the National Bureau of Statistics of China. The eastern region includes Hebei, Zhejiang, Shandong, Liaoning, Jiangsu, Fujian, Guangdong, and Hainan (seven provinces). The central region contains six provinces, including Heilongjiang, Jiangxi, Hubei, Shanxi, Jilin, Anhui, Henan, and Hunan. The western region includes eleven provinces, which are Inner Mongolia, Xinjiang, Guangxi, Guizhou, Shaanxi, Qinghai, Tibet, Sichuan, Yunnan, Gansu, and Ningxia. Table 7 shows the regression results divided by east, middle, and west. We found that compared with the central and western regions, the BCS only played a significant role in promoting the level of digitization in the eastern region. We explained this finding by analyzing the demand and supply sides. The eastern region has a more developed economy, so the demand for digitalization is higher. The eastern region also has more convenient infrastructure and available financing, so the conditions for digital transformation are relatively suitable. Therefore, after the implementation of the BCS, the digital level of local enterprises in this region significantly improved.

**Table 7:** Regression results for eastern, central, and western China.

	(1)	(2)	(3)
	East	Middle	West
Treat×Post	0.0488***	0.0255	0.0134
	(3.3474)	(0.9835)	(0.3607)
*N*	13116	3629	2768
*R* ^2^	0.3057	0.2730	0.2254
Control	YES	YES	YES
FirmFE	YES	YES	YES
YearFE	YES	YES	YES
F	86.5958	25.7756	20.2220

Note: T-statistics for each coefficient are provided in parentheses.

*, **, and *** indicate 10%, 5%, and 1% significance levels, respectively.

Data source: authors’ calculations. Before the analysis of heterogeneity, we standardized all variables.

(2) Subgroup based on ownership. In a developing country such as China, state-owned enterprises still account for a large proportion of the total. Different from private enterprises, this type of enterprise has a natural connection with the government, and pursues the maximization of social welfare in operation. From the results reported in Table 8, the regression coefficient of network infrastructure construction on the digitalization of non-state-owned enterprises was positive at the 1% significance level, and the regression coefficient for state-owned enterprises was not significant. Furthermore, the *2019 China Digital Enterprise White Paper* shows that state-owned and private enterprises have different perceptions of the importance of digital strategies. State-owned enterprises lack the motivation to digitalize mainly due to the lack of mature digitalization plans. Without the support of complete digital transformation, state-owned enterprises are unwilling to quickly implement digital transformation. Therefore, non-state-owned companies can benefit more from this infrastructure construction, as they have better basic digital capacities and can consequently make better use of this pilot policy to improve their digital level than their state-owned counterparts.

**Table 8:** Regression results for different enterprise ownership types in China.

	(1)	(2)
	State-owned	Non-state-owned
Treat×Post	-0.0017	0.0549***
	(-0.0866)	(4.3167)
*N*	7423	13581
*R* ^2^	0.2543	0.3058
Control	YES	YES
FirmFE	YES	YES
YearFE	YES	YES
F	26.1557	96.3267

Note: T-statistics for each coefficient are in parentheses.

*, **, and *** indicate 10%, 5%, and 1% significance levels, respectively.

Data source: authors’ calculations. Before the analysis of heterogeneity, we standardized all variables.

(3) Subgroup based on the three kinds of input factors. By comparing the relative importance of the input factors, we divided the enterprises into three categories: labor-, capital-, or technology-intensive. These enterprises, having different factor densities, had different resource endowments, transformation needs, and motivations. From the regression results in Table 9, we found that only the digital level of the technology-intensive enterprises was positively affected by the BCS, with a coefficient value of 0.0374. The coefficient values of labor- and capital-intensive enterprises were also positive, and the latter was on the verge of significance (the t-values in the parentheses are close to the critical value). Therefore, we think that technology-intensive companies can better use digital infrastructure to generate internal and external coordination. This is also the ultimate goal of the government in promoting the BCS.

**Table 9:** Regression results for different enterprise types.

	(1)	(2)	(3)
	Labor intensive	Capital intensive	Technology intensive
Treat×Post	0.0551	0.0293	0.0374***
	(1.2508)	(1.6556)	(3.4124)
*N*	1993	5322	5848
*R* ^2^	0.3560	0.2223	0.3083
Control	YES	YES	YES
FirmFE	YES	YES	YES
YearFE	YES	YES	YES
F	289.0841	101.3047	918.3374

Note: T-statistics for each coefficient are provided in parentheses.

*, **, and *** indicate 10%, 5%, and 1% significance levels, respectively.

Data source: authors’ calculations. Before the analysis of heterogeneity, we standardized all variables.

(4) Subgroup based on five digitalization sources. Digitization mainly includes big data, cloud computing, artificial intelligence, and blockchain technology, which are usually abbreviated as ABCD [43]: A, artificial intelligence; B, block chain; C, cloud computing; and D, big data. These four technologies have different degrees of dependence on networks. Enterprise digitization is more related to the application of digital technology, so we also distinguished digital technology applications. The results of the group regression are shown in Table 10. We found that BCS implementation positively affected different types of digitalization. Among the five sources, companies were experiencing the most rapid growth in artificial intelligence and cloud computing, both of which strongly rely on networks. In addition, the application of digital technology was the main development direction [44]. As mentioned above, the BCS has not only strengthened the construction of network facilities but also stimulated the application of digital technology. Promoting network construction is important for the digitalization of enterprises.

**Table 10:** Regression results for different digital resources.

	(1)	(2)	(3)	(4)	(5)
	lnai	lnblock	lncloud	lndata	lnapp
Treat×Post	0.0909***	0.0048**	0.0760***	0.0223***	0.0732***
	(3.8022)	(2.5635)	(3.4835)	(2.7687)	(2.6986)
*N*	21295	21295	21295	21295	21295
*R* ^2^	0.1862	0.0161	0.1389	0.0448	0.1997
Control	YES	YES	YES	YES	YES
FirmFE	YES	YES	YES	YES	YES
YearFE	YES	YES	YES	YES	YES
F	8.0332	4.0724	8.1103	5.3570	71.8541

Note: T-statistics for each coefficient are provided in parentheses.

*, **, and *** indicate 10%, 5%, and 1% significance levels, respectively.

Data source: authors’ calculations.

## Conclusion and implication

With the rapid development of the digital economy, the digital transformation of enterprises has increasingly become the focus of social attention. However, owing to the positive externality of digitization, organizations are facing challenges in finishing the digital transformation process on their own. The government must play a key role in this. As such, in this study, we used data from China’s listed companies from 2010 to 2020 to determine whether the implementation of the BCS has empowered enterprises to digitize. We found the following: (1) The BCS has significantly promoted the growth in corporate digitalization. This conclusion remained valid after eliminating possible endogenous problems and controlling government digital subsidies and differences at the city level. (2) The BCS has heterogeneously impacted the increase in corporate digitalization. The improvement in network infrastructure has mainly promoted the digitization of non-state-owned enterprises, which have a higher demand for the infrastructure, technology-intensive enterprises with stronger application capabilities, and enterprises in the eastern region with richer resources. In addition, the implementation of the BCS has promoted the use artificial intelligence and cloud computing, which rely on the Internet.

Through the above findings, we obtained the following recommendation: First, in the process of promoting the digitalization of enterprises, the government should actively play a role by strengthening their support of new infrastructure for enterprises. Second, the BCS has affected various organizations in different ways. While strengthening infrastructure, the supply of digital service providers should be concurrently increased to better satisfy organizations’ transformation demands. Finally, because of regional and industry variances in company digitization, a customized development plan is an essential future path.

However, this study is not without limitations, which need further consideration. (1) The granular data were limited. Differences occurred in the execution of the BCS among the pilot cities. Because we could not obtain specific information for each city, such differences within the group were not well-identified. We expect that better statistics will be available in the future to support related research. (2) As a major infrastructure country, China has recently adopted hundreds of city-level pilot projects, such as photovoltaics, new energy vehicles, and sponge cities. The results of the BCS, being one of them, will be influenced by other pilot projects. We attempted to prevent endogeneity and urban characteristics from influencing the results, but did not analyze the impact of other pilot initiatives implemented during the same time period. Furthermore, our findings are limited to China and may not be applicable elsewhere.

## Supporting information

S1 Dataset (RAR)
